# Observations on Some Popular Remedies of German Practitioners

**Published:** 1843-07

**Authors:** George Lefevre


*Observations on some Popular Remedies of German Practitioners.*—By Sir George Lefevre, M.D., &c.—German therapeutics hold a middle rank of action between the French and the English, being more energetic than the former, and less so than the latter. The Germans boast of a simplicity of prescription, and have a horror of contrarieties, carrying this to a ludicrous nicety and an unmeaning orthodoxy. Thus, a solution of sulphate of magnesia in an infusion of roses, or the combination of a laxative with an astringent, meets with the severest criticism, from those who profess as much abhorrence of a *contresens* in prescriptions as nature does of a vacuum.

There is much to commendate in simplicity of prescription, and the multifarious ingredients which formerly entered into the British recipe have not undeservedly merited the stigma of *farrago* applied to the latter by continental practitioners. Dr. Paris long since pointed out the error, and has done much to rectify it. The study of pharmaceutic chemistry and the superior education of the general practitioner of modern days have almost accomplished this desideratum; still we do meet with combinations of materials which would be better for a little sifting.

Unless in form of decoctions, which contain certainly the essence of a large proportion of the vegetable kingdom, and are favorite remedies, a German prescription rarely boasts otherwise of more than two or three ingredients. Nothing is considered to be inert unless it be distilled water, and if active remedies are administered in a variety of other menstrua, the latter are not chosen indifferently, but with a specific view, and to perform a part in the operation of the whole. One carminative water cannot be substituted with impunity for another, it being granted that each has a specific action on the system. In respect to the influence of minute doses the Germans countenance the practice of homœopathists. Their ideas (well or ill-founded as they may be) of infinitesimal doses are illustrated in the preparation of a decoction much esteemed for the cure of eruptions which disfigure female beauty, and which is administered freely in the spring. It is composed of sarsaparilla, dulcamara, tops of the pine, beet-root, buds of the beach tree, &c.; but the most active ingredient is a small piece of glass of antimony tied up in a muslin bag, and boiled for a limited time only in the decoction, which is supposed to be impregnated by it, although it may have lost no weight in the operation.

With respect to active remedies they are generally exhibited in their most simple form. Calomel is generally given in powdered sugar. Blue-pill is rarely, if ever, prescribed, and I have not met with any German practitioner practically acquainted with the hyd. c. cretâ.

When it is the object to introduce mercury by slow degrees into the system, the bichloride is always employed. I must state that I was much disappointed in the effects of our common English preparation of blue-pill in northern latitudes, and I must attribute the failure to some influence in the climate, and not to any fault in the preparation, for I imported it myself from the best London chemists, so that during the latter period of my sojourn in St. Petersburg I invariably employed the bichloride in the treatment of equivocal complaints.

In no climates do these diseases more readily yield to the influences of mercury than in those of northern latitudes, and more particularly in the winter season. The equable temperature of the apartments prevents the patients from catching cold, and the dryness of the atmosphere allows, under certain restrictions, of moderate exposure to the open air. It is positive that the cure is prolonged by external exposure, but it is equally true that the constitution on the whole suffers less than when subjected to close confinement.

I shall not at present agitate the question of the non-mercurial treatment of these complaints, which I may describe at some future period; I shall at present speak of the bichloride, placing it at the head of the list of popular German remedies, upon which I propose making a few observations.

In primary affections, where the use of mercury is indicated, the patient is put upon low diet, and but a small quantity of animal food, and that of the plainest kind, is permitted. Wine and spirituous liquors are interdicted by the generality of practitioners, but some allow of a moderate quantity of the former, upon the principle of its preventing the debilitating effects of nausea, and mitigating diarrhœa and tormina. Coffee is looked upon as too stimulating, and all condiments in the preparation of the patient's food are prohibited. As a general position, the more simply the body is nourished the more powerfully will specific remedies act upon the disease under which it labors, and the theory, rational in itself, is supported by facts. The patient is generally purged, and advised to take a warm bath previous to the commencement of the course, and the latter is generally repeated once a week during the treatment.

The following is the usual manner of administering the bichloride. I shall quote the practice of Dr. Salomon, whose successful operation of the ligature of the common iliac has placed him high in the ranks of surgical eminence:

℞ *Hydrarg. bichlor.*, gr. iij; *solve in aquâ*
*fontan.*, gtt. ij.
*Ext. glycyrrhiz*, ℨj;
*Opii puri*, gr. iij. Misce et distribue in pilulas xviij æquales.

The patient commences by taking one pill the first night and one the following morning. The same the second night and morning. Two pills the third night, one in the morning. The same the fourth and fifth night. The sixth night two pills, and two the following morning. Three pills on the eigth night.

The dose is never pushed beyond a grain in the twenty-four hours, and seldom more than two-thirds of a grain are given daily. If things go on on well, and the disease feels the influence of the remedy, the dose is again reduced, so that towards the end of the third week no more than the sixth of a grain is taken daily. A pint of the simple decoction of sarsaparilla is drank during the course of the day. Black-wash upon finely scraped lint is the local application, and it is rare that a cure is not effected in the course of three weeks, or after the administration of from ten to twelve grains of the bichloride. The treatment is terminated by an aperient, and two or three warm baths. Such is an outline of the practice which I have seen very generally adopted, and which latterly I employed myself, and I have met with no cases of secondary symptoms where it has been conscientiously followed by the patient.

In some constitutions, which rebel against a continued course of mercury, the hydriodate of potash is substituted for a time.

It is administered in large doses, from ten to thirty grains, three times per day. If the local condition of the part improves, and the constitutional irritation ceases, the mercury is again resorted to for the final cure. The Germans seldom combine the bichloride with the hydriodate of potash. In pseudo-syphilitic cases the treatment is confined to the administration of the latter with sarsaparilla. In decided secondary symptoms small doses of the bichloride, long continued, with the decoction.

There is a difference of opinion among German practitioners as regards the advantages or disadvantages of cutaneous perspiration during the administration of mercury. Some advise that the patient should be confined to a room heated to 60° of Fahr., and combine sarsaparilla with the bichloride, from its sudorific properties; whereas a distinguished physician in Vienna, who has acquired celebrity for the treatment of syphilis, particularly under its secondary forms, insists upon a cool surrounding atmosphere, asserting that if the pores of the skin are kept open, mercury loses half its power.

*The Muriate of Ammonia.*—Previous to my sojourn in Russia I had never seen this remedy employed, otherwise than in the form of lotion. I have within the last fourteen years had much experience of it as an internal medicine, and have had very convincing proofs of its efficacy, or even preference over other salts, in a variety of complaints. I have stated that the tubercular consumption is a rare disease in high northern latitudes; but pleuritic affections, subacute and congestive inflammation of the lungs and their envelopes, are very frequent maladies. These are the effects of vicissitudes of temperature on the breaking up of the winter, and go under the name of *refroidissement*, a term which implies more than our English phrase of catching cold.

In cases of serous and mucous congestion, where the inflammation does not run so high, I have seen this salt employed with the most beneficial effects. In pleuritic affections it is usual to combine tartarised antimony with it, and the following is the formula generally adopted:—

℞ *Mur. ammoniæ*, ʒj;

*Antimon. tartar*, gt. ij;

*Decoc. glycyrrhiz*, ℥viij;

*Syrup. altheæ*, ℥j M.

A tablespoonful of this is given every two hours, and continued in spite of the nausea and vomiting which the first dose or two may occasion.

Administered *per se*, and prescribed with the same view as nitrate of potash, I should, as far as my own experience is concerned, give it a decided preference. I would not exalt it to the dignity of a specific, nor attribute all its virtues to its deobstruent power, as most of its advocates do. It acts slightly upon the kidneys, and expectoration is often facilitated, but not always so, and cases treated by it *progress* to cure without decided critical evacuation.

In cases of congestion of the mucous membranes, in chronic sore throat, with elongated uvula and flabby state of tonsils and parts about the fauces, it is very beneficial. In that state of the mucous membrane of the stomach caused by the action of a variety of medicines which gives rise to the anorexia accompanying convalescence, I have seen it employed with happy results. The tongue loses its pallor, and acquires a healthier appearance from its use, and it paves the way advantageously for the more decided tonics in the convalescence after gastric fever.

In some forms of uterine disease accompanied by discharges it is also useful. Its salt, unpleasant taste is best disguised in a decoction of liquorice-root, or a solution of the extract in water.

*The Nitrate of Soda.*—The Germans consider this salt as possessing great antiphlogistic powers, and more active than the nitrate of potash; it is indicated in cases of acute inflammation of the lungs and pleura, and supersedes the muriate of ammonia, which is given in subacute and congestive inflammations. I have not the testimony of experience to warrant any preference it has over the nitrate of potass. The Germans still retain the names of natron for soda, and kali for potash. They say natron nitricum, &c.

*Bitter Water.*—This is a favorite remedy, and is much used in St. Petersburg; its action is mild and speedy, and when taken in tumbler doses early in the morning seldom fails to purge gently; its taste is mawkish, but much less nauseous than the solutions of Epsom or Glauber salts. Previous to a commencement of a course of mineral waters, it is customary to administer a few doses of this to prepare the way for them. Pregnant women employ this remedy with very great advantage, for it produces all the desired effects without any griping.

*The Muriate of Soda*, in the form of brine, in which cucumbers are preserved, is a most popular aperient. A small watery, seedy cucumber is pickled in brine, to which a very small proportion of vinegar and some leaves of aromatic plants are added. Thousands of barrels are prepared annually, and serve as salad for rich and poor during the winter. The liquor, impregnated with the rind of the cucumber and the aromatic herbs, is drank in tumbler doses, and is more popular even than the bitter water.

*Aqua Lauro-cerasi.*—This preparation is in much and deserved esteem in Germany. I am surprised to find it so rarely employed by London practitioners; it is highly serviceable in spasmodic affections, and is what the French style *calmant* in the most extensive signification of the term. It is prussic acid *drawn mild,* but it is considered to be more available, and, indeed, is almost used to the exclusion of it by continental practitioners. It is a safer preparation; the dose may be increased from ten to sixty drops, and a patient may be trusted with a phial of it. There is a great advantage in administering certain remedies, *per se;* by diffusing them in mixtures made palatable by syrup, &c., decompositions take place, as, for example, chlorine water, which is decomposed by sugar.

The druggists in St. Petersburg are compelled to retail all medicines which are poisonous in small doses in *dark blue bottles,* and all the labels are of the same colored paper. A piece of paper, upon which is printed "*for external use,*" is pasted upon the foot of the phials containing liniments, lotions, &c. This is a very sensible plan, and worthy of imitation.

The aqua lauro-cerasi deserves a trial. It is most useful in spasmodic affections of the stomach, in hypochondriac uneasiness, in hysteria, combined with pain about the uterus. Some mental affections from over excitement are much relieved by it, I have found it very useful in calming the pain arising from the passage of inspissated bile, and small biliary calculi. Whether all these effects are to be derived from the hydrocyanic acid, I have not sufficient experience; if they are equally certain I still prefer the aqua lauro-cerasi as a more available remedy, which may be administered, *per se.*

*Phosphoric Acid.*—I should say that this is given in all those cases where sulphuric and nitric acid are employed in Britain, and that Germans consider it a better tonic than the two latter. It is prescribed in its solid form in pills, but more frequently in the liquid state.

*Chlorine Water.*—This is a very favorite remedy in putrescent fevers, and in cases of ulcerated sore throat which assume a putrid form. It is useful in gargles, and I have employed it with much success in St. Petersburg, where sore throats are very prevalent, owing to the alternations of extreme cold and moisture; I have prescribed it also in form of mixture in camphor julep, or some simple menstruum. Sugar decomposes it.

*Arnica Montanum.*—This has been introduced into the British Pharmacopœia since I left the country. Whether it has justified this honor by the estimation in which it is held by the profession I am unable to state. With the Germans it is classed among *sacred* remedies; its virtues are extolled throughout two pages of the "Pharmacopœia Ruthensis." I have been much disappointed in its effects as far as I have been conversant with it. It is generally used in those cases where strychnine is indicated, but is much less efficacious and more uncertain in its operation. I have seen it administered in large doses without producing any sensible effects.

*Extracts.*—These are very freely used by German practitioners, and the two general favorites are the ext. taraxaci, and the ext. graminis (triticum repens). The former is employed in all hepatic affections, jaundice, dropsy, &c. The latter is used as a mild tonic and deobstruent, in the convalescence from gastric fevers. Perhaps no two remedies are so frequently prescribed as the two in question; a great merit is their indigenous growth. The Russians, as far as drugs are concerned, patronise home produce; they have a prejudice against taking such as do not grow in the same quarter of the globe in which they reside, and question the influence of Asiatic products upon European constitutions, unless the European happen to be located in Asia. The domestic medicines of the people, the roots, the herbs, the simples, are also much in vogue with the higher classes, from the circumstance of their indigenous growth.

*Indigo.*—This remedy I have seen administered in cases of epilepsy, hysteria, and in all the convulsive affections of children, from such as are the result of teething to those which are caused by effusion on the brain. I have ever protested against the idea of its exerting any beneficial power in such cases, and have discontinued my attendance where a continuation of its use has been insisted upon to the exclusion of more active remedies. It is, nevertheless, a very popular remedy with German practitioners. The

*Treatment of Erysipelas* by the latter is altogether confined to antiphlogistic remedies administered internally, and to dry applications externally. The application of anything in liquid form to the part is most severely reprobated, and a practitioner who would apply a cooling lotion to an erythematous inflammation of the leg would risk the censure of the board of medicine (the physicant). There is a great and prevailing fear amongst German physicians of repelling anything in form of eruption, and should this have taken place it seems sufficient to explain the production of any subsequent malady at any remote period. The blood once contaminated by the retrocession of an *ausschlag* (eruption), will require quarts of decoctions to purify it. I have heard of dangerous fevers attributed to the use of Gowland's lotion, as puerperal mania and swelled leg are always ascribed to the absorption of milk into the blood. The *Rose* is looked upon with a jealous eye both as to immediate and future consequences, and a patient who has once suffered from erysipelas is hardly considered out of jeopard for the rest of his life. If any means have been used to cure the complaint by local applications, then the danger is quadrupled. Metastasis from the extremities to the head is held up *in terrorem*. The treatment in incipient stages is confined to laxatives, sudorifics, and nauseating doses of antimony, and the part is dusted with starch, powder, or chalk and camphor, and the whole limb is inclosed in wadding. If the head be attacked a mask of this cotton wadding is made to envelop it, apertures being left for the eyes, mouth, and nostrils, so that the patient looks like a Spanish inquisitor; all means are employed to divert to the extremities, and the pediluvium with mustard-flour, sinapisms, and dry cupping are resorted to. No narcotics are permitted at any stage of the complaint, not even to procure sleep when all unpleasant symptoms have vanished. The powder with which the parts are dusted goes by the name of *anti-erysipelatous powder of Berlin.*

*Tinct. Digitalis Æther.*—This is a very useful preparation, and a convenient mode of administering the remedy. The leaves of the foxglove are macerated in ether in lieu of rectified spirit. The nauseating properties of the digitalis are counteracted by the stimulant power of the menstruum, and in cases of serous effusion, where it is desirable to increase the action of the absorbents and to determine to the kidneys, this preparation seems to combine these advantages, without the nausea and exhaustion which frequently accompanies the use of the simple tincture. From five to twenty drops is its range of dose.—*London Lancet*, April 29, 1843.

*Advantages of Tapping the Brain in Hydrocephalus.*—Mr. Butcher, in a late number of the "Dublin Medical Journal," advocates strongly the employment of the trocar to penetrate the lateral ventricles of the brain in certain cases of hydrocephalus; and the operation may, he considers, be performed with a successful result at a much later period than it has usually been resorted to. No doubt the serous fluid effused into the ventricles becomes at an after period absorbed, and the patient dies from the structure of the brain becoming secondarily involved; but Mr. Butcher says,—

"That ramollissement does not come on till a later period than is generally supposed, I have verified by dissection. In eight cases that I have examined of acute hydrocephalus, where the head was unusually large, and where the children were carried off by infectious diseases, not in any of those cases was there softening or ramollissement of the structure of the brain, though the lateral ventricles were enormously distended with fluid."

A case is reported illustrative of the good effects obtained by tapping. It was that of an infant, sixteen months of age, the head of which "was almost transparent, and presented in different situations patches where the ossific matter was not deposited, its place being supplied by the common integuments. The sutures were very considerably separated, being from half to three-quarters of an inch asunder. The head measured in the fronto-occipital circumference thirteen inches and a half; from ear to ear across the vertex, nine inches; round the chin and across the vertex, nine inches and three quarters.

The operation was conducted as follows:

"The integuments having been divided with a lancet, a trocar was introduced about three-quarters of an inch external to the mesial line, near the junction of the edge of the fontanelle with the margin of the parietal bone. The instrument being inclined a little inwards, in this manner it was directed into the lateral ventricle of the right side; no sooner was the stilet withdrawn than the fluid flowed out freely through the canula. The quantity drawn off amounted to eleven ounces. After the discharge of so large a quantity of fluid, the head lost its tension and globular form, and became so flaccid as to allow the remaining quantity of water to gravitate backwards, giving the head a very elongated appearance. To support the parts a double-headed roller was applied loosely, the edge of the incision being previously brought together with adhesive plaster. The child sunk very low immediately after the operation; however, after some time, the urgent symptoms were suppressed. From this period up to four weeks after, every thing was doing remarkably well; the head filled again, but not to one-half of its original size. The operation was again performed on the left side, in the same situation as on the right. The child began visibly to improve; no convulsions whatever occurred after the first operation; four weeks more elapsed, and the head was not near the size it presented before the second operation; so much was it lessened that another operation was deferred for a future period."

Though the result was so eminently successful, it is lamentable to think that the patient should afterwards have succumbed through ill-treatment.

"Ten weeks had elapsed; the child was free from convulsions; the eyes were quite sensible to light, and the little patient did not exhibit any lethergetic symptoms. In four days after he began to get uneasy and restless; three more had not passed by when he was seized with convulsions that terminated in death. I was immediately called to see the child, and having accused the mother of neglecting it in some way she acknowledged to have given it wine regularly for five days before, stating, as her reason for doing so, 'She thought it would hasten the cure, and not do him any harm; he was so much better.' "—*London Lancet*, April 29, 1843.

